# Fast Simultaneous Determination of Eight Sudan Dyes in Chili Oil by Ultra-High-Performance Supercritical Fluid Chromatography

**DOI:** 10.1155/2019/3731028

**Published:** 2019-01-10

**Authors:** Yunjia Yang, Jing Zhang, Jie Yin, Yi Yang

**Affiliations:** ^1^Beijing Key Laboratory of Diagnostic and Traceability Technologies for Food Poisoning, Beijing Center for Disease Prevention and Control, Beijing 100013, China; ^2^School of Public Health, Capital Medical University, Beijing 100069, China

## Abstract

A rapid and simple ultra-high-performance supercritical fluid chromatography (UHPSFC) with photodiode array (PDA) method was developed and validated for simultaneous determination of eight Sudan dyes in chili oil. In particular, a pair of isomer, Sudan red B and Sudan IV, was included in the analysis. After being diluted with dichloromethane, the analytes were separated on an Acquity UPC^2^ HSS C18 SB column with gradient elution using CO_2_ as the mobile phase and acetonitrile/methanol (v/v, 45/55, containing 0.1% formic acid) as the organic modifier. Analytes were quantified by external calibration curves over ranges of 0.5–50 mg/L, with correlation coefficients above 0.999. The method gave recoveries of the target compounds (spiked at levels of 1, 5, and 25 mg/kg) ranging from 82.6 to 108.3%, with intraday and interday relative standard deviations of less than 8.0% and 8.6%, respectively. The limits of detection (LODs) and the limits of quantification (LOQs) for eight dyes were from 0.10 to 0.30 mg/kg and 0.30–1.00 mg/kg, respectively. This method was applied for the analysis of chili oil samples collected from the supermarket in Beijing. This validated that the UHPSFC-PDA method provides a useful strategy for the simultaneous determination of Sudan dyes in chili oil for routine analysis.

## 1. Introduction

Sudan dyes are lipophilic azo dyes that are commonly used as industrial dyes and biochemical reagents. They have been banned for use as food additives because of their carcinogenicity. The remarkable characteristic of Sudan dyes is the fact that they are chromophoric azo groups, which can be reduced by mammalian and microbial enzyme systems. It has been documented that the reduction of azo bonds is an important factor that contributes to the toxicity, mutagenicity, and carcinogenicity of Sudan dyes [1–5]. Sudan dyes are classified as category 3 carcinogens, and its two metabolites, including *ortho*-aminoazotoluole (*o*-AOCB) and 4-aminoazobenzene (4-AOB), are classified as category 2 carcinogens by the International Agency for Research on Cancer [6]. Sudan I, II, III, and IV were completely banned for use in food by the EU [7], China, (GB2760-2014) [8], and the USA [9]. However, because of their low cost and wide availability, Sudan dyes are illegally used in food occasionally, especially in chili and chili products.

**Figure 1:**
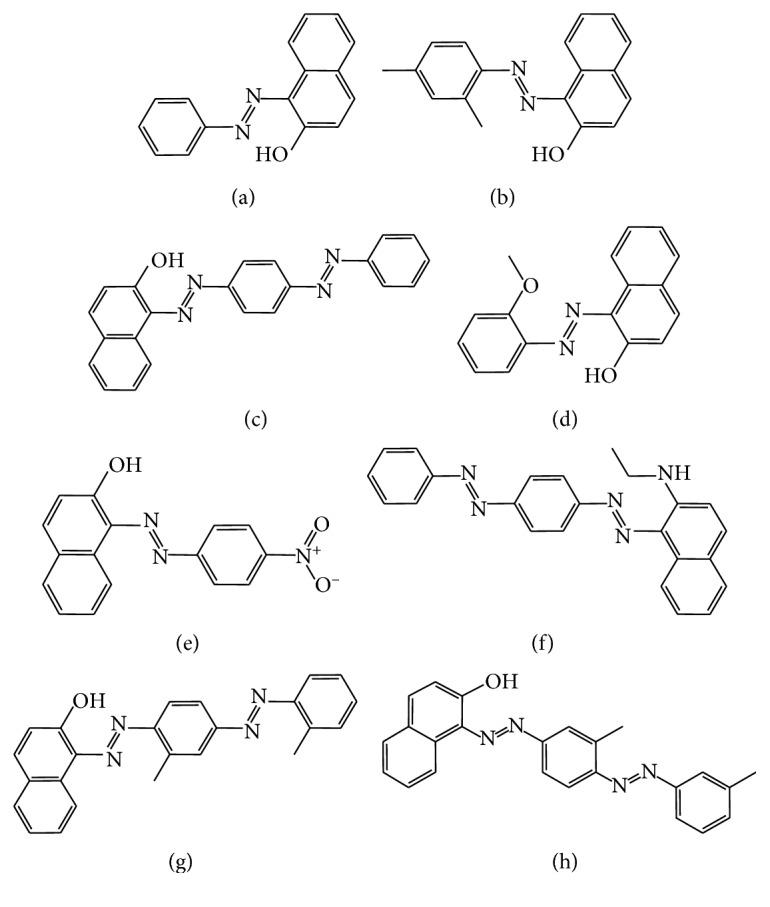
Chemical structure of eight Sudan dyes. (a) Sudan I. (b) Sudan II. (c) Sudan III. (d) Sudan red G. (e) Para red. (f) Sudan red 7B. (g) Sudan IV. (h) Sudan red B.

In the past decade, in light of the increasing and widespread illegal uses of Sudan dyes in food around the world, many studies have been developed to identify and quantify Sudan dyes in foodstuff [10–14]. Previous studies have adopted micellar electrokinetic chromatography and capillary electrophoresis to detect Sudan dyes in foodstuff [15–17]. More recently, liquid chromatography mass spectrometry (LC-MS) or liquid chromatography tandem mass spectrometry (LC-MS/MS) has been used for the analysis of Sudan dyes because of its high sensitivity and selectivity [18–21]. However, compared with those expensive instruments, the photodiode array detector (PDA) is still the most commonly used detection instrument in routine analysis due to its simple operation at the analysis step and low cost. On the contrary, performing PDA on an HPLC system for the determination of Sudan dyes is really a challenging task since they include a group of lipophilic chemicals having more similarities in molecular structure and some of them are even isomers. In this study, a new analytical method was utilized for the separation of Sudan dyes in food: ultra performance convergence chromatography (UPC^2^). It integrates supercritical fluid chromatography (SFC) and LC technologies and shows many advantages including high efficiency, low cost, reduced solvent consumption, and sensitivity with the aid of a special column compacted with sub-2 *μ*m particles, which makes its application in routine analysis more attractive [22, 23]. Supercritical fluid is a state with liquid-like density and gas-like viscosity and will function much better than a liquid solvent as an extractant or as a mobile phase in extraction and chromatography. Although supercritical fluid was first used as a mobile phase in 1962, SFC was gradually developed into a truly useful separation method during the latter half of the 1990s [24]. Today, SFC has been widely used in analytical chemistry and the pharmaceutical industry, especially in chiral separation and analysis of lipophilic compounds due to its higher theoretical plate values than LC and its lipophilic nature [25–27]. Khalikova et al. [28] developed an ultra-high-performance supercritical fluid chromatography method for determination of illegal dyes in food. But in that study, the chili oil sample was not mentioned. As shown in Figure 1, Sudan IV and Sudan red B are a pair of isomers, and the only difference was the position of the methyl group. Therefore, it was difficult for Sudan IV and Sudan red B to be completely separated by HPLC. Considering the different retention mechanism compared to that of the reversed-phase HPLC system, UHPSFC was selected in order to realize the simultaneously complete separation of eight Sudan dyes including Sudan IV and Sudan red B.

In addition, for the pretreatment of chili oil samples, gel permeation chromatography (GPC) is the popular method to purify and enrich Sudan dyes because the oily matrix has a high lipid content, and Sudan dyes are fat-soluble compounds [29, 30]. The large volume of uses of organic solutions and time-consuming procedures is the main shortcomings of the GPC method. It was exciting that the abundant amount of lipids did not disturb the separation of Sudan dyes because the separation mechanism is similar to normal phase LC. Another advantage of SFC is the excellent compatibility of the SFC mobile phase with the common extraction solvents used for nonpolar compounds. Therefore, the GPC cleanup procedure is no more necessary to determine the Sudan dyes in chili oil by SFC separation technology. As mentioned above, the aim of the present work was to develop and optimize UHPSFC separation coupled with the PDA detection method for the simultaneous determination of eight Sudan dyes in chili oil.

## 2. Materials and Methods

### 2.1. Chemicals and Reagents

Sudan I, Sudan II, Sudan III, Sudan IV, Sudan red 7B, Sudan red G, Sudan red B, and Para red were obtained from Dr. Ehrenstorfer GmbH (Augsburg, Germany). Water was prepared by a Milli-Q ultrapure water system (Millipore, Bedford, MA.). HPLC-grade acetonitrile, methanol, dichloromethane, and isopropanol were purchased from Dikma (Lake Forest, CA). Formic acid was obtained from Acros Organics (Morris Plains, NJ).

### 2.2. Standard Solution Preparation

The 1000 mg/L stock standard solutions of eight Sudan dyes were prepared in methanol, respectively. They were combined in the mixed working standard solution and further diluted also with methanol.

### 2.3. Sample Collection and Preparation

Fifteen commercial chili oil samples with different brands were purchased from local supermarkets in Beijing. These samples were stored at 4°C before analysis using the newly developed method.

2.0 mL chili oil sample was added into a 15 mL centrifuge tube and then 2.0 mL dichloromethane was added into the sample. After vortex mixing for 30 seconds, the extract was centrifuged at 10,000 rpm for 10 min, and the supernatant was directly analyzed by UPC^2^.

### 2.4. Instrument and Chromatographic Conditions

The ultra-high-performance supercritical fluid chromatography (UHPSFC) system Acquity UPC^2^ (Waters, Milford, USA), coupled with an Acquity PDA detector, was used to separate and determine eight Sudan dyes. The separation was performed by Acquity UPC^2^ HSS C18 SB column (100 × 3.0 mm, 1.7 *μ*m, Waters). Gradient elution was performed using CO_2_ (A) and methanol-acetonitrile (55/45, v/v) containing 0.1% formic acid (B) as modifier at flow rate 2.0 mL/min. The gradient schedule of the mobile phase was started with 95% A, followed by a linear gradient to 85% A in 1 min, and held for 2 min. At 3.5 min the percent of A was increased to 95% linearly, and the column was equilibrated for 1.5 min with 95% A until the next injection. The temperature of the autosampler and the column oven were set at 4°C and 45°C, respectively. The back-pressure regulator (BPR) pressure was typically set to 2000 psi. The injecting volume was 2.0 *μ*L. Methanol was used as a needle-wash solvent. The PDA detection was performed at 200–600 nm with reference wavelength at 600–650 nm. The extraction wavelengths for quantitative analysis were set as follows: 480 nm for Sudan I, Para red, Sudan II, and Sudan red G; 500 nm for Sudan III, Sudan red B, and Sudan IV; 520 nm for Sudan red 7B. Peak identification was done by comparing the retention times and absorption spectra of samples with dye standard solutions.

## 3. Result and Discussion

### 3.1. Influence of Stationary Phase

**Figure 2:**
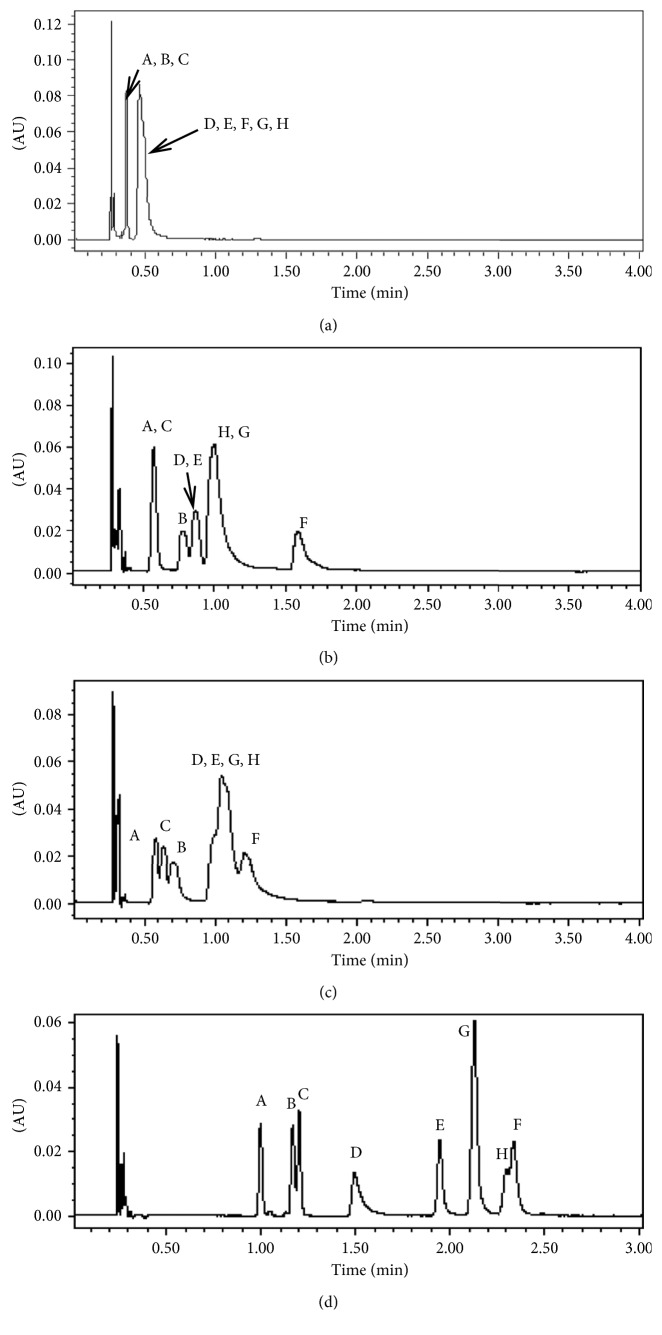
The chromatograms (480 nm) of eight Sudan dyes processed by Acquity UPC^2^ BEH (a), Acquity UPC^2^ BEH 2-EP (b), Acquity UPC^2^ CSH FP (c), and Acquity UPC^2^ HSS C18 SB (d): (A), Sudan I; (B), Para red; (C), Sudan II; (D), Sudan red G; (E), Sudan III; (F), Sudan red 7B; (G), Sudan red B; (H), Sudan IV.

The choice of the stationary phase in SFC is a key step in optimizing chromatographic conditions. In this paper, four available commercial columns including Acquity UPC^2^ BEH (1.7 *μ*m, 3.0 × 100 mm), Acquity UPC^2^ BEH 2-EP (1.7 *μ*m, 3.0 × 100 mm), Acquity UPC^2^ CSH Fluoro-Phenyl (1.7 *μ*m, 3.0 × 100 mm), and Acquity UPC^2^ HSS C18 SB (1.8 *μ*m, 3.0 × 100 mm) were tested. These columns differed both in selectivity and polarity. The experiments were performed under the same chromatographic conditions by gradient elution using 5% to 15% of methanol as an organic modifier within 3 minutes at 2.0 mL/min, 55°C, and 2000 psi on all stationary phases. As shown in Figure 2, the lowest retentive capability and resolution for all eight target analytes was with the BEH (bridged ethyl hybrid) stationary phases. The retention times of all of the target analytes by BEH 2-EP were greater than the BEH column because the retentive capability was increased due to the modification of 2-ethylpyrimidine. However, Sudan I and Sudan II, Sudan red G and Sudan III, and Sudan red B and Sudan IV were not separated. Therefore, the columns of BEH and BEH 2-EP were both not suited to detect eight target Sudan dyes. The CSH FP (CSH Fluoro-Phenyl) stationary phase could increase the retention capability for the aromatic compounds by *π*-*π* interactions between the phenyl groups on the stationary phase and the aromatic groups in the analytes. But in this study, the resolution and retention of target analytes were still not satisfactory even if they were better than BEH and BEH 2-EP. It was exciting that all eight Sudan dyes can be effectively separated by an Acquity UPC^2^ HSS C18 SB column, especially for the two isomers, Sudan IV and Sudan red B achieved baseline separation. Sudan IV and Sudan red B are two isomers with different positions for the methyl group bonded to the benzene ring. As shown in Figure 1, the 2′,2″-methyl is at the *ortho*- position of the azo group in Sudan IV, while the 3′,3″-methyl group is at the *meta*- position of the azo group in Sudan red B. Sudan IV and Sudan red B were completely separated on the Acquity UPC^2^ HSS C18 SB column, and the retention time of Sudan IV was higher than that of Sudan red B. It was speculated that different retention behaviors of Sudan IV and Sudan red B was related to the different steric hindrance, although the explicit separation mechanism was unclear.

### 3.2. Influence of Organic Modifier

**Figure 3:**
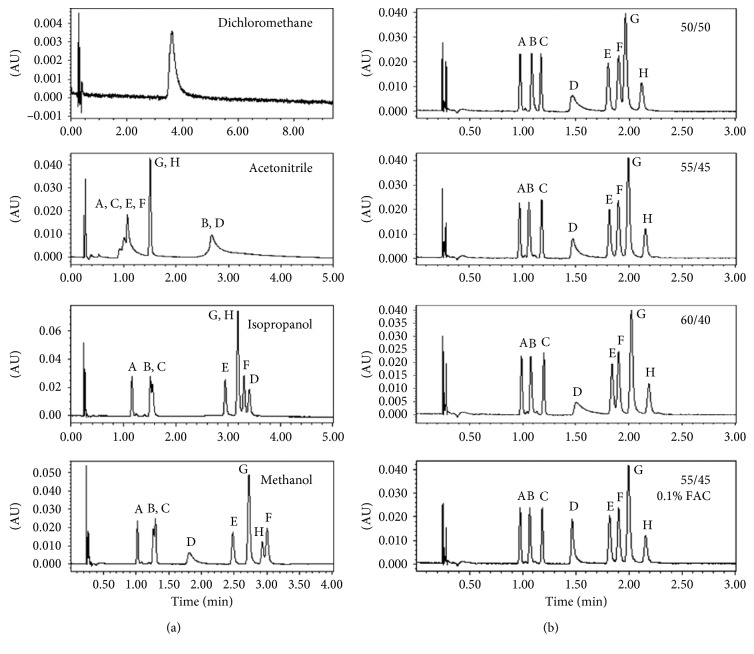
The chromatograms (480 nm) of eight Sudan dyes processed by different organic modifiers (a) and different ratio of methanol/acetonitrile (v/v) as organic modifier (b): (A), Sudan I; (B), Para red; (C), Sudan II; (D), Sudan red G; (E), Sudan III; (F), Sudan red 7B; (G), Sudan red B; (H), Sudan IV.

In SFC, various organic solutions as organic modifiers, such as methanol, ethanol, acetonitrile, isopropanol, and dichloromethane, were often added into the CO_2_ in order to increase the polarity of the mobile phase and improve peak shape or retention. According to the above study, Para red and Sudan II, and Sudan red 7B and Sudan IV were not completely separated with methanol as the organic modifier. In this study, methanol, acetonitrile, isopropanol, and dichloromethane were investigated as organic modifiers by the same elution procedure of isocratic elution with 20% of methanol at 2.0 mL/min. In SFC, the order of elution ability for the organic modifiers was methanol > acetonitrile > isopropanol > dichloromethane. As shown in Figure 3(a), dichloromethane was not suitable for use as an organic modifier for Sudan dyes separation by SFC because of its lower elution strength, pool selectivity, and low resolution (the retention time for all target dyes was the same at 3.85 min). Although the chromatographic resolution was remarkably increased by acetonitrile or isopropanol, the two isomers, Sudan red B and Sudan IV, were not completely separated. But it is fortunate that Sudan red B and Sudan IV were completely separated by methanol. However, the resolution of Para red and Sudan II were also lower than 1.0, and the peak of Sudan red G was tailed. In order to improve resolution of Para red and Sudan II, acetonitrile was added to methanol. As shown in Figure 3(b), acetonitrile changed the order of elution for Sudan IV and Sudan red B and increased the resolution of Sudan IV and Sudan red B. The optimal ratio of acetonitrile and methanol was selected as 45/55 (v/v) due to the optimal resolution for all target dyes. However, one deficiency in the above chromatography conditions is the peak width of Sudan red G was too large. To improve the peak shape, 0.1% formic acid was added in combination with organic modifier, and the results indicated that the formic acid appears to be an effective additive (Figure 3(b)).

### 3.3. Influence of Pressure, Temperature, and Flow Rate

For SFC chromatography, the temperature of column is an important chromatography condition because the elution strength of the mobile phase is related to the density of fluid, which is negatively correlated to temperature. In this study, different column temperatures including 35°C, 40°C, 45°C, 50°C, 55°C, and 60°C were investigated. Increasing column temperature was found to strengthen the retention of Para red but no remarkable change for other dyes. When column temperature was set as 45°C, the optimal resolution between Sudan I and Para red, and between Para red and Sudan II was acquired. Therefore, 45°C was selected as the analysis condition. Under the tested conditions, there were no substantial changes in selectivity of separation for all Sudan dyes when the pressure and flow rate were changed.

### 3.4. Method Validation

#### 3.4.1. Specificity

**Figure 4:**
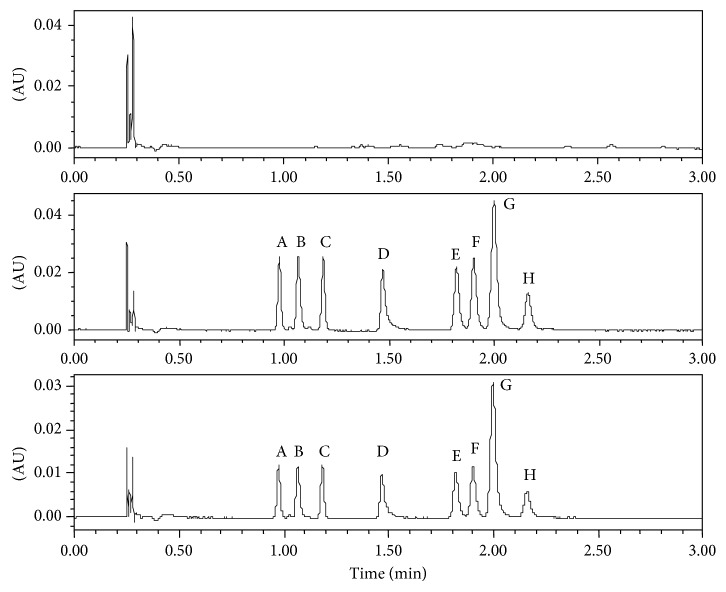
The chromatograms (480 nm) of eight Sudan dyes of a blank sample solution (a), standard solution (b), and spiked sample solution (c): (A), Sudan I; (B), Para red; (C), Sudan II; (D), Sudan red G; (E), Sudan III; (F), Sudan red 7B; (G), Sudan red B; (H), Sudan IV.

In this study, the specificity was evaluated by comparing the chromatograms of mobile phase blank, standard solution, and spiked sample solution. The 10 *μ*L of blank sample solution, standard solution, and spiked sample solution were injected into the HPLC system separately, and the chromatogram results are shown in Figure 4. It can be observed that there was no coeluting peaks at the retention time of eight target dyes in the blank sample. Therefore, the specificity of the method was confirmed.

#### 3.4.2. Linearity and Range

The linearity of method was evaluated using working standard mixtures of eight dyes over a range of 0.5–50 mg/L. Integrated peak areas of the selected absorbing wavelength for each dye were used to construct eight-point calibration curves, which were applied for quantification. From the regression analysis, the slopes and intercepts of linear equations for target dyes were obtained as shown in Table 1. The correlation coefficients (*r*^2^) of the calibration curves were greater than 0.9994 for all target dyes. The above results indicated that there were a good linear relationship between the concentration of eight dyes and area under the peak.

#### 3.4.3. Limit of Detection (LOD) and Limit of Quantification (LOQ)

The LOD and LOQ were estimated by analyzing 10 control samples and calculated as 3 and 10 times the standard deviation of the signals from the blank matrix, respectively. For each dye, the LOD and LOQ calculated in the chili oil sample are shown in Table 1.

#### 3.4.4. Accuracy

The accuracy of the method was evaluated by assaying recovery of the fortified chili oil samples at three concentration levels. As presented in Table 2, average recoveries of each dye ranged from 82.6% to 108.3% at three spiked levels (1 mg/kg, 5 mg/kg, and 25 mg/kg). The interday and intraday RSD% were 1.6–8.0% and 4.0–8.6%, respectively. These results demonstrated that the accuracy of this method is suitable for routine monitoring purposes.

#### 3.4.5. Precision

The precision of the method is expressed as the relative standard deviation. In this study, the relative standard deviation of RT, peak area, and measured result of eight dyes was investigated with 10 replications. The results showed that the RSD of RT, peak area, and measured result was lower than 5%. Therefore, the good precision of method was confirmed.

#### 3.4.6. Robustness

The robustness was determined by analyzing the same samples under variety of conditions. Extraction time of vortex mixing (30 s, 60 s, 90 s, and 120 s) and centrifugation time (5 min, 10 min and 15 min) were compared. As a result, extraction time and centrifugation time did not have significant effects on the method.

#### 3.4.7. Solution Stability

In this study, the stability of solution was investigated for 24 h at room temperature. As the result, the recoveries of the same standard solution in 0 h and 24 h at room temperature were all higher than 98%, and the RSD was all lower than 2%. The result indicated a good stability of standard solutions for 24 h at room temperature.

### 3.5. Application to Real Samples

The developed and validated method was used to analyze fifteen chili oil samples with different brands purchased from local markets. The results indicated that none of the target compounds were detected in the chili oil samples analyzed.

## 4. Conclusions

In this study, a rapid and reliable method has been established for the simultaneous determination of eight dyes in chili oil samples by ultra-high-performance supercritical fluid chromatography. Acceptable accuracy and precision values were obtained for all eight dyes. This method can be used for the routine surveillance of dyes in chili oil.

## Figures and Tables

**Table 1:** The linear range, slope, intercept, correlation coefficient, LOD, and LOQ of eight Sudan dyes.

Dyes	Linear rang (*μ*g/mL)	Slope	Intercept	*r* ^2^	LOD (mg/kg)	LOQ (mg/kg)
Sudan I	0.5–50	2.72 × 10^3^ ± 51.4	−1.78 × 10^2^ ± 3.4	0.9995	0.15	0.50
Para red	0.5–50	3.17 × 10^3^ ± 39.2	−4.60 × 10^2^ ± 8.5	0.9997	0.15	0.50
Sudan II	0.5–50	2.76 × 10^3^ ± 48.5	−1.18 × 10^2^ ± 2.3	0.9998	0.15	0.50
Sudan red G	0.5–50	3.72 × 10^3^ ± 29.7	−1.18 × 10^2^ ± 1.9	0.9994	0.20	0.60
Sudan III	1.0–50	3.52 × 10^3^ ± 40.3	−5.87 × 10^2^ ± 10.6	0.9995	0.20	0.60
Sudan red 7B	0.6–50	4.09 × 10^3^ ± 80.7	−6.86 × 10^2^ ± 10.8	0.9995	0.15	0.50
Sudan red B	0.3–50	8.40 × 10^3^ ± 77.6	2.91 × 10^2^ ± 5.7	0.9997	0.10	0.30
Sudan IV	1.0–50	2.58 × 10^3^ ± 19.8	−4.48 × 10^2^ ± 6.0	0.9999	0.30	1.00

**Table 2:** The recovery, intraday, and interday RSD of eight Sudan dyes.

Dyes	Spiking level (mg/kg)	Mean recovery (%)	Intraday RSD (%)	Interday RSD (%)
Sudan I	1	96.6	6.7	8.6
	5	102.5	5.2	5.4
	25	98.2	3.8	6.2

Para red	1	99.5	4.0	5.4
	5	97.3	6.4	4.3
	25	96.9	4.2	6.6

Sudan II	1	108.3	2.1	5.0
	5	101.5	1.6	5.4
	25	103.7	4.1	4.8

Sudan red G	1	98.5	3.2	4.1
	5	99.2	2.0	4.0
	25	106.4	2.5	4.7

Sudan III	1	95.5	5.8	4.9
	5	94.6	3.2	6.0
	25	98.3	6.2	5.8

Sudan red 7B	1	93.8	6.3	7.0
	5	96.5	3.5	6.6
	25	97.9	4.4	5.9

Sudan red B	1	82.6	6.9	8.1
	5	88.9	8.0	8.6
	25	90.1	4.2	6.7

Sudan IV	1	96.3	4.6	5.1
	5	98.9	5.2	6.1
	25	105.2	4.0	5.8

## Data Availability

The data used to support the findings of this study are included within the article.
